# Pharmacogenomic and pharmacogenetic-guided therapy as a tool in precision medicine: current state and factors impacting acceptance by stakeholders

**DOI:** 10.1017/S0016672315000099

**Published:** 2015-06-01

**Authors:** GREGORY P. HESS, EILEEN FONSECA, RACHEL SCOTT, JESEN FAGERNESS

**Affiliations:** 1 Symphony Health Solutions, Horsham, PA, USA; 2 Leonard Davis Institute, University of Pennsylvania, Philadelphia, PA, USA; 3 Independent Researcher, Harleysville, PA, USA; 4 Genomind, Inc., King of Prussia, PA, USA; 5 Geneist, LLC, Cambridge, MA, USA

## Abstract

Pharmacogenetic/pharmacogenomic (PGx) testing is currently available for a wide range of health problems including cardiovascular disease, cancer, diabetes, autoimmune disorders, mental health disorders and infectious diseases. PGx contributes important information to the field of precision medicine by clarifying appropriate treatments for specific disease subtypes. Tangible benefits to patients including improved outcomes and reduced total health care costs have been observed. However, PGx-guided therapy faces many barriers to full integration into clinical practice and acceptance by stakeholders, whether practitioner, patient or payer. Each stakeholder has a unique perspective on the role of PGx testing, although all are similarly challenged with demonstrating or appraising its cost-to-benefit value. Coverage by insurers is a critical step in achieving widespread adoption of PGx testing. The acceleration of adoption of precision medicine in general and for PGx testing in particular will be determined by how quickly robust evidence can be accumulated that shows a return on investment for payers in terms of real dollars, for clinicians in terms of patient clinical responses, and for patients in terms of economic, health and quality of life outcomes. Trends in PGx testing utilization and uptake by payers in real-world practice are discussed; the role of pharmacoeconomics in assessing cost-effectiveness is highlighted using a case study in psychiatric care, and several issues that will affect adoption of PGx testing in the United States (US) over the next few years are reviewed.

## 1. Introduction

Pharmacogenomic and pharmacogenetic testing, which identify genetic variants that can help predict drug efficacy and/or toxicity, are important contributors to the field of precision medicine by clarifying appropriate treatments for specific disease subtypes. Recognizing potential differences in terminology, depending on whether single drug–gene interactions or drug responses from multiple genes are referred to, we will use PGx as an abbreviation inclusive of both pharmacogenomic and pharmacogenetic approaches in this review.

Response rates of patients to medications ranges widely by therapeutic class, from ~80% for analgesics to ~25% for oncology therapeutics (Spear *et al.*, 2001). In addition to the varied response rates, adverse drug reactions (ADRs) also vary widely and are of significant clinical concern. ADRs are estimated to occur in excess of 2 million events in the US each year and result in over 100 000 deaths annually (Lazarou *et al.*, 1998). Although genetic variation seldom accounts for all of the differences seen in patient treatment responses and ADRs, the objectives of PGx testing are to utilize genomic information to tailor pharmacotherapy to the patient's predicted treatment response in order to improve drug efficacy, real-world effectiveness and safety. Genetic information (such as DNA sequence, gene expression and copy number) is used to explain inter-individual differences in drug metabolism (pharmacokinetics) and responses (pharmacodynamics). Expectations are that PGx testing will target drug therapies to the appropriate patients in order to maximize benefits and to minimize harms, as well as costs. The use of PGx testing has already begun for many drugs in development, and the marketing of drugs currently available may soon suggest or require that PGx testing is done before a patient ever begins a treatment regimen.

PGx testing is currently available for a wide range of health problems including cardiovascular disease, cancer, diabetes, autoimmune disorders, mental health disorders and infectious diseases. Tangible benefits to patients are currently being observed on a daily basis, including improved outcomes and reduced total health care costs. However, PGx-guided therapy faces many barriers to full integration into clinical practice and acceptance by stakeholders, whether practitioner, patient or payer.

## 2. Purpose of this review

Each stakeholder has a unique perspective on the role of PGx testing, although all are similarly challenged with demonstrating or appraising its cost-to-benefit value. Coverage by insurers is a critical step in achieving widespread adoption of PGx testing, and the current and future landscape relative to reimbursement is a focus throughout this review. In the following sections, trends in PGx testing utilization and uptake by payers in real-world practice are discussed, the role of pharmacoeconomics in assessing cost-effectiveness is highlighted using a case study in psychiatric care, and several issues that will affect adoption of PGx testing in the US over the next few years are reviewed. The purpose of this review is to increase the understanding of the current state of PGx testing, discuss key factors impacting its acceptance, and to foster additional research and evidence generation in the field.

## 3. Current real-world PGx-testing practice

Over 140 US Food and Drug Administration (FDA)-approved drugs have PGx information in their labelling (FDA, 2014 *b*) that refers to pharmacodynamic relationships, such as identification of a specific gene that correlates with a drug mechanism of action, and/or pharmacokinetic mechanisms that describe drug metabolism effects on efficacy and ADRs. For a limited number of drugs, labelling mandates PGx testing and specific actions are taken based on the PGx information (examples, primarily highlighting the area of oncology, are provided in Table 1(*a*)). Where PGx testing has been identified as necessary for the safe and effective use of the corresponding therapy, companion diagnostic tests have been approved (premarket approval) or cleared (equivalent to marketed device) by the FDA (FDA, 2014 *a*) (examples provided in Table 1(*a*)). In these situations, the cost for testing indicated by the FDA is generally reimbursed by most insurance plans (Hresko & Haga, 2012; Graf *et al.*, 2013). This is well illustrated in the field of oncology. Since many cancers are not viewed as a single disease, but rather as a group of several subtypes, each with a distinct molecular signature, identifying the genomes of the malignancy and of the patient can aid in effective and safe treatment.

**Table 1.** Examples of drugs with PGx biomarker and use, labelling status, companion PGx test, and payer coverage.

Drug (FDA, 2014 *b*)	Therapeutic area (FDA, 2014 *b*)	PGx Biomarker/allele (FDA, 2014 *b*)	Use (FDA, 2014 *b*)	Labelling status (FDA)	Companion Diagnostic Test (FDA, 2014 *a*)	Payer coverage (Epstein *et al.*, 2009)
A. Labelling mandates PGx testing						
Cetuximab, panitumumab	Oncology	EGRF, KRAS	Efficacy	Mandatory testing required by the FDA to confirm patients have EGFR-positive colorectal cancer with wild-type KRAS. Drugs may be ineffective in patients with tumors expressing KRAS mutation (Bristol-Myers Squibb, 2013 *a*; Amgen, 2014)	*therascreen* KRAS RGQ PCR Kit (Qiagen Manchester Ltd.) DAKO EGFR PharmDx Kit (Dako North America, Inc.)	Generally covered and reimbursed
Vemurafenib, plaparib	Oncology	BRAF V600E	Efficacy	Mandatory testing required by the FDA for the mutation prior to drug use for melanoma (vemurafenib) or ovarian cancer (plaparib) (Astra Zeneca, 2015; Genentech, 2015)	BRACAnalysisCDx™ (Myriad Benetic Lab., Inc.) COBAS 4800 BRAF V600 Mutation Test (Roche Molecular Systems, Inc.)	Generally covered and reimbursed
Imatinib	Oncology	BCR-ABL translocation c-KIT (CD117)-positive unresectable tumors	Efficacy	Mandatory testing required by the FDA for confirmation of disease and selection of patients for which the drug is indicated (Novartis, 2014)	DAKO C-KIT PharmDx (Dako North America, Inc.)	Generally covered and reimbursed
Trastuzumab	Oncology	HER2	Efficacy	Mandatory testing required by the FDA for HER2-over-expressing cancers prior to treatment (Genentech, 2014)	INFORM HER-2/NEU (Ventana Medical Systems, Inc.) PATHVYSION HER-2 DNA Probe Kit (Abbott Molecular Inc.) PATHWAY ANTI-HER-2/NEU (4B5) Rabbit Monoclonal Primary Antibody (Ventana Medical Systems, Inc.) NSITE HER-2/NEU KIT (Biogenex Laboratories, Inc.) SPOT-LIGHT HER2 CISH Kit (Life Technologies, Inc.) Bond Oracle Her2 IHC System (Leica Biosystems) HER2 CISH PharmDx Kit (Dako Denmark A/S) NFORM HER2 DUAL ISH DNA Probe Cocktail (Ventana Medical Systems, Inc.) HERCEPTEST (Dako Denmark A/S) HER2 FISH PharmDx Kit (Dako Denmark A/S)	Generally covered and reimbursed
Crizotinib	Oncology	ALK	Efficacy	Mandatory testing required by the FDA to confirm the presence of lymphoma kinase (ALK) mutation prior to drug use (Pfizer, 2014)	VYSIS ALK Break Apart FISH Probe Kit (Abbott Molecular Inc.)	Generally covered and reimbursed
Ivacaftor	Pulmonary	CFTR *G551D* variant	Efficacy	If the patient's genotype is unknown, FDA requires an FDA-cleared mutation test to detect the presence of the *G551D* mutation (Vertex, 2014)	Not required since most patients have genotyping performed at diagnosis	Generally covered and reimbursed
B. Labelling recommends PGx testing						
Abacavir	Infections disease (HIV)	HLA-B*5701	Hypersensitivity reactions	Boxed warning of increased risk in patients with HLA-B*5701. Prior to initiating therapy with abacavir, screening for the HLA-B*5701 allele is recommended. Patients tested positive should not receive abacavir (Aidsinfo NIH, 2013)	FDA approved/cleared tests are available, but companion testing not required	Coverage varies by insurer
Carbamazepine	Neurology	HLA-B*1502, HLA-A 3101	Stevens-Johnson syndrome (SJS) and toxic epidermal necrolysis (TEN)	Warning of increased risk for increased risk of SJS and TEN in patients with HLA-B*1502. Patients from high-risk regions (e.g. Southeast Asia) should be screened for HLA-B*1502 before starting carbamazepine (Novartis, 2009)	FDA approved/cleared tests are available, but companion testing not required	Coverage varies by insurer
Clopidogrel	Cardiology	Defective CYP2C19 alleles (e.g. CYP2C19*2, CYP2C19*3)	Efficacy	Information of possible reduced effectiveness in CYP2C19 homozygotes/intermediate and poor metabolizers (Bristol-Myers Squibb, 2013 *b*)	FDA approved/cleared tests are available, but companion testing not required	Insurers may cover a single test depending on indication
Codeine	Anesthesiology	Duplicated or amplified CYP2D6 alleles	CNS depression	Information regarding patients who are ultra-rapid metabolizers secondary to the CYP2D6*2XN genotype and who could have much higher morphine concentration resulting in increased risk for CNS symptoms related to overdose, even when treated with standard doses (Roxane, 2009)	FDA approved/cleared tests are available, but companion testing not required	Insurers may cover a single test depending on indication
C. Labelling for PGx testing is informational only						
Rovustatin	Endocrinology	*SLCO1B1*	Toxicity (myopathy)	Labelling states that the impact of this polymorphism on efficacy and/or safety of rosuvastatin has not been clearly established (Astra Zeneca, 2014)	FDA approved/cleared tests are available, but companion testing not required	Considered investigational/not covered
Warfarin	Cardiology and Hematology	*CYP2C9* and *VKORC1*	Efficacy and toxicity (bleeding)	Labelling provides dose recommendations according to *CYP2C9* and *VKORC1* genotypes, but does not recommend routine testing (Bristol-Myers Squibb, 2014)	FDA approved/cleared tests are available, but companion testing not required	Considered investigational/not covered

Other drugs have labelling with PGx specific guidelines for a patient subtype but don't have a required companion PGx diagnostic. For example, ivacaftor is approved for cystic fibrosis therapy specifically for patients with the *G551D* mutation, which occurs in 4% of patients (Clancy *et al.*, 2014). The majority of cystic fibrosis patients have a mutation panel performed at the time of diagnosis as an existing standard of care; labelling guidelines require an FDA approved mutation test in cases where the genotype is not known (Vertex, 2014). In either case, costs are typically reimbursed (Hresko & Haga, 2012; Graf *et al.*, 2013). Examples of other drugs with recommended, but not mandated, PGx guidelines are shown in Table 1(*b*). While these examples are not comprehensive, they illustrate the diversity of drugs and therapeutic areas where the FDA has approved or cleared PGx tests, but the companion testing is not required prior to prescribing. Coverage for testing in these cases varies widely by insurers.

**Fig. 1.**
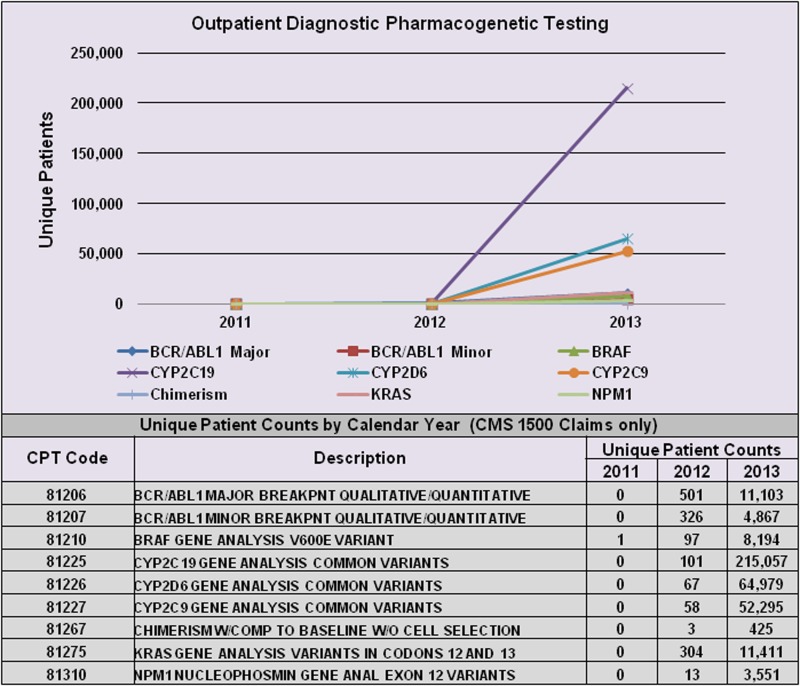
Increase in PGx testing from 2011–2013. Figure shows the change in unique patient counts for selected assays from 2011 through 2013. On 1 January 2012, Medicare requested that claims for Molecular Pathology Procedures reflect both the existing CPT ‘stacked’ test codes that are required for payment, and the new single CPT test code. Patient counts are based on CMS 1500 claims and are courtesy of Symphony Health Solutions private practitioner medical claims database. CPT, Current Procedural Terminology.

However, PGx-specific labelling for many drugs is informational only, and testing is neither mandated nor recommended prior to prescribing. Table 1(*c*) provides examples of two such cases (rovustatin and warfarin). In these cases, PGx testing has generally not been endorsed by expert committees since the supporting evidence has been judged statistically insufficient, and consequently, insurers will not pay for it (Hresko & Haga, 2012; Graf *et al.*, 2013). This is particularly relevant for tests related to genotyping for metabolizing enzyme polymorphisms for use with drugs having narrow therapeutic ratios and/or serious toxic effects. Figure 1 illustrates the growth of PGx testing in this area between 2011 and 2013 using data available from a private practitioner medical claims database (Symphony Health Solutions). FDA approval or clearance is not a requirement, so many are laboratory developed tests (i.e. offered and used within a single laboratory) that do not undergo FDA review. However, there is currently new FDA draft guidance in review that may change the regulatory oversight of laboratory developed tests (FDA, 2015 *b*). For acceptance and reimbursement of these tests by payers, comparisons with standard methods of assessing therapeutic drug safety and efficacy are needed, as well as subsequent demonstration of value. Since factors other than genetic polymorphisms (including race, gender, epigenetic factors and lifestyle choices) can have a significant effect on drug pharmacokinetics and pharmacodynamics, it remains unclear whether PGx testing would eliminate the need for simultaneous use of other methods of therapeutic drug monitoring.

In the area of therapeutic drug monitoring a recent example with potentially broad applications has been the use of PGx testing to guide warfarin anticoagulation therapy for the prevention of thromboembolic events. Two genes have been identified that play a role in outcomes related to warfarin therapy: *CYP2C9* is a gene for an enzyme that is primarily responsible for the metabolism of warfarin, and *VKORC1* codes for the warfarin drug target. The current warfarin label acknowledges that dose requirements are influenced by *CYP2C9* and *VKORC1* and states that genotypic information, when available, can assist in selecting the starting dose, but does not recommend routine PGx testing for determining initial or maintenance doses. Several diagnostic tests are cleared by the FDA for monitoring of warfarin anticoagulation (FDA, 2015 *a*). However, the lack of standardization of these tests in detecting the relevant *CYP2C9* variants, lack of understanding of the role of specific *VKORC* haplotypes and varying result turnaround times (1 to 8 hours) have raised skepticism regarding their decision-making utility (Rosove & Grody, 2009). Randomized controlled clinical trials examining use of PGx-guided warfarin dosing compared with current clinical data monitoring approaches have shown inconsistent results in stabilization of treatment responses, reducing the number of necessary office visits or decreasing risk of bleeding events. Consequently, consensus guidelines (e.g. American College of Chest Physicians, American College of Medical Genetics), do not advise use of genetic testing to guide warfarin dosing (Flockhart *et al.*, 2008; Guyatt *et al.*, 2012). As a result, most payers (including the Centers for Medicare and Medicaid Services (CMS)) are reluctant to reimburse for testing for PGx-guided warfarin therapy. However, it is unclear what role patient compliance with treatment contributes to the inconsistent efficacy and health outcomes, and studies addressing non-genomic factors' influence on treatment outcomes are critical to justify modification to standards of care and reimbursement decisions.

The drug clopidogrel, an anti-platelet therapy, is another example where genotype-specific labelling guidelines do not meet the indeterminate threshold for standard of care testing. *CYP2C19* genotyping can determine normal, poor, intermediate, rapid and ultra-rapid metabolizers of clopidogrel in order to identify patients who may benefit from non-standard, ‘recommended’ dosing or have better responses to an alternative drug. In 2010, the FDA added a boxed warning to clopidogrel labelling alerting patients and health care professionals that the drug can be less effective in people who cannot metabolize the drug to convert it to its active form, and modified the warning to include guidance on data suggesting adverse cardiovascular event risks for acute coronary syndrome (ACS) patients with the *CYP2C19* genotype following a percutaneous coronary intervention (PCI) (Bristol-Myers Squibb, 2013 *b*). However, the boxed warning does not mandate genetic testing, is not specific on the exact patient profile to benefit from genetic testing, and is vague on alternative treatment approaches in poor metabolizers. Consensus guidelines issued by the American Heart Association (AHA) and American College of Cardiology (ACC) express concerns about the lack of outcomes data documenting the benefit of routine genotyping and concluded the available literature did not support *CYP2C19* genotyping for all patients being prescribed clopidogrel (Holmes *et al.*, 2010). AHA and ACC guidelines to date support *CYP2C19* genotyping in ACS patients at high risk for poor outcomes after PCI or in patients receiving antiplatelet therapy for whom the genotype poses potential risk for reduced antiplatelet efficacy (Scott *et al.*, 2013). Genotyping for one *CYP2C19* polymorphism in patients prescribed clopidogrel is reimbursed by some payers (Hresko & Haga, 2012; Graf *et al.*, 2013).

Genetic testing has also been considered to mitigate the risk of serious adverse events with statins, which are generally safe and well tolerated and among the most widely prescribed medications (e.g. almost 50% of adults 65 years and older have statin prescriptions (CDC, 2013). Myopathy is the most common side effect and can result in life-threatening rhabdomyolysis with muscle damage and acute renal injury. Although mild myalgias may not result in any physical harm, they threaten patient adherence to statin therapy. The incidence of statin-associated myopathy varies between 1 and 5% in clinical trials, with a higher incidence observed in clinical practice (Thompson *et al.*, 2003). There is a heritable component to the risk for statin-induced myopathy and the role for PGx testing in guiding statin pharmacotherapy is evolving, particularly for the solute carrier organic anion transporter family, member 1B1 (*SLCO1B1*) gene. Routine PGx testing for statin therapy is not recommended by current guidelines (Ramsey *et al.*, 2014; Talameh & Kitzmiller, 2014) nor covered by most payers. Additional studies are needed to determine clinical utility and cost-effectiveness of PGx testing in patient populations given factors including the type of statin (as pharmacokinetic profiles for each statin are unique), the dose and concomitant use of other drugs (Sorich *et al.*, 2013; Talameh & Kitzmiller, 2014).

Inclusion of informational PGx tests by the FDA on the revised labels of many drugs without clear guidance on dosing recommendation and/or therapeutic alternatives by PGx result has led to confusion among providers, who are eager for guidance around an emerging technology with which they are largely unfamiliar. Professional organizations are attempting to educate and guide physicians on drug selection and dosing for drugs with PGx informational labelling. For example, the Clinical Pharmacogenetics Implementation Consortium has issued dosing guidelines taking into consideration patient genotype for warfarin, opioids, abacavir and other drugs with and without PGx informational labelling (CPIC, 2015). However, although these organizations can provide some level of guidance, challenges remain as there are limited nationally and internationally accepted standards around dosing decisions. Continued education of health care providers regarding the impact of metabolizer status and frequency of variants in a given population is needed to fully leverage PGx testing and information.

## 4. Pharmacoeconomic analyses to demonstrate value: case study of PGx-guided psychiatric intervention

The use of PGx testing needs to exhibit clinical, economic and quality of life benefits; demonstrating one or all of these values is critical to adoption among stakeholders. In addition to reducing side effects and ADRs, goals of PGx testing include lowering costs and improving patient compliance. When PGx testing results guide treatment decisions, positive treatment responses will be more quickly attained, which in turn, in theory, reduces the indirect and direct costs associated with failed treatment trials and ADRs. As long as the PGx testing costs and practitioner time for interpretation are lower than these indirect and direct costs, the net result is cost savings. In addition, patient quality of life improves when treatment failure and ADRs are reduced. While indirect costs are important considerations for providers and patients, payers are most influenced by evidence of direct impact, thus research has a multifaceted challenge of proving value on several levels. One well-established approach for determining value is to assess both costs and outcomes within pharmacoeconomic analyses. There have been relatively few published cost-effectiveness analyses of PGx interventions. The following example demonstrates how PGx-guided psychiatric intervention was associated with increased compliance with therapy and direct cost savings (Fagerness *et al.*, 2014). While cost-effectiveness data are not the only information needed to influence acceptance of PGx testing by stakeholders, payers are particularly interested in these data, and the ability to use real-world claims and clinical databases is an efficient method to demonstrate utility in the absence of randomized clinical trials.

Known pharmacodynamic and pharmacokinetic variations exist that impact responses to common psychiatric medications (Burroughs *et al.*, 2002). An estimated two-thirds of patients with depression will not respond adequately to first line treatment and more than one-third of patients will become treatment resistant (Kessler *et al.*, 2003; Souery *et al.*, 2006; Warden *et al.*, 2007). Genetic variations affect efficacy and tolerability of psychotropics as a result of variable drug disposition, metabolism and transport (Evans & McLeod, 2003; Kao *et al.*, 2011; Porcelli *et al.*, 2011; Salloum *et al.*, 2014). Many antidepressants and antipsychotics show differences in plasma drug levels as a result of cytochrome P450 polymorphishms (e.g. *CYP2D6* and *CYP2C19*) (Kirchheiner *et al.*, 2004; Tansey *et al.*, 2013). The Genecept™ Assay (Genomind) is a laboratory validated test used to analyse variations in both pharmacodynamic and pharmacokinetic genes associated with treatment response, side effects, metabolism, tolerability and overall efficacy of psychiatric medications (Kato & Serretti, 2010; Lencz *et al.*, 2010; Zhang *et al.*, 2010; Psychiatric GWAS Consortium Bipolar Disorder Working Group, 2011; Bhat *et al.*, 2012; Porcelli *et al.*, 2012). Despite its commercial use for over 4 years in more than 30 000 patients, the Genecept Assay is considered ‘experimental’ by many insurers. Typical to challenges in this field, while this assay has accumulated multiple levels of data arguably supporting its utility, the evidence to overcome this label is rigorous, but paradoxically not well defined.

One such piece of evidence is a retrospective, observational study that assessed patient claims data to measure direct health costs as a result of patient and clinician access to genetic information during psychiatric treatment selection guided by the Genecept Assay (Fagerness *et al.*, 2014). Genetic test results and pharmacy and medical claims were integrated at the patient level. Propensity score matching (PSM) was used to identify case and control patients to reduce potential sources of bias (Baser, 2006; Austin, 2010; Luo *et al.*, 2010; Austin, 2011). PSM adjusted for covariates of patient demographics, payer type, medical data and treating practitioner's specialty, and identified 111 case and 222 matched control patients from pools of over 1000 case and 100 000 control patients. Patient's highest adherence levels pre-index vs. post-index were compared (index = date when genetic test results were available for tests and similar calendar date for controls). An observed increase in adherence to therapy in cases was associated with a significant *decrease* in overall costs associated with outpatient activity. Although pharmacy costs for cases increased relative to controls, likely due to consistent medication fills concomitant with improved medication adherence, the overall use of medical services decreased, and the relative cost savings over a 4-month follow-up period was 9·5%, or $562 per patient (Fagerness *et al.*, 2014).

While randomized controlled trials remain the gold standard for clinical investigation, this example demonstrates how retrospective observational studies can use real-world clinical data to assess whether PGx-guided therapy can improve patient outcomes. This approach can assist in building the clinical and economic evidence base for PGx testing in order to provide information to stakeholders. To be effective in influencing uptake of PGx testing, health care providers will benefit from increasing awareness of the data on cost-effectiveness and economic utility of PGx.

## 5. Acceptance of PGx-guided therapy by insurers

### (i) Increasing the evidence base of PGx testing

An important driver for uptake of PGx testing is insurance coverage and reimbursement.

A variety of factors are considered by insurers in formulating medical coverage policies for PGx testing, including availability of clinical guidelines, current use by physicians, patient interest and cost-effectiveness (Meckley & Neumann, 2009) .The most consistent determining factor in coverage and reimbursement is conclusive evidence linking the use of the PGx test with health outcomes (Cohen *et al.*, 2013). Government and private insurers rely on evidence of the impact of PGx testing on clinical and economic outcomes compared to an appropriate real-world alternative intervention in a prospective study. Currently, few PGx tests have evidence to support their clinical utility and consequently, they are considered experimental by most insurance plans and denied coverage. This behavior is consistent with insurers' approach to reimbursing drugs and medical procedures.

To avoid being considered experimental, providers of PGx tests need to make sure they perform robust studies and publish in peer-reviewed literature; i.e., conform to the norms established by insurers and standards groups. While randomized controlled trials remain the gold standard, it is possible to demonstrate clinical utility with other study models. As shown in the previous section, the use of claims and clinical data can establish benefits through the efficiency of data collection and the ability to measure direct and some indirect costs.

The CMS has established policies designed to bridge the divide between evidence-based coverage standards and emerging PGx technologies. The coverage with evidence development (CED) status is conferred by the CMS on promising PGx tests (as well as other technologies) (Tunis & Pearson, 2006). This designation encourages use of the test in clinical trials by linking provisional coverage for promising technologies to the requirement for patient participation in a registry or clinical trial. CMS has assigned CED designation to PGx testing for warfarin, for example (CMS, 2014 *a*). The designation encourages collection of real-world data to assess clinical utility and economic outcomes and may influence other payers to provide reimbursement for promising PGx tests in a similar manner.

### (ii) Novel coverage and reimbursement models

Reimbursement levels for diagnostic tests, including PGx testing, are regulated by the CMS Clinical Laboratory Fee Schedule. A new coding system and reimbursement plan enacted by CMS in 2014 introduced analyte-specific codes to identify individual tests (CMS, 2014 *b*). PGx testing has been especially impacted by the new model. The ‘unstacking’ of diagnostic test codes has resulted in less bundling of tests and provided pricing transparency that has resulted in greater scrutiny by payers of PGx tests (Deverka, 2009). The prior billing system used stacked codes and many payers typically did not have visibility into which analyte a test examined or how practitioners used it. The increased transparency has provided the means and ability for payers to assess the many individual PGx tests on the market, although information regarding how a test is performed and how clinicians use the information is still insufficient. For example, in the gene *CYP2D6*, there are well over 20 variations that can influence how the gene will behave in any given person. However, with the new coding system, an insurance company does not know if one or 20 variants have been tested. This could lead to many issues for patients, as well as interpretation problems for the providers. Payers are hesitant to reimburse for a PGx test if the information generated does not change practitioner and patient practices or if results of PGx tests are ignored. As a result, insurers are turning to new coverage and payment models such as performance-based and risk-sharing models (Carlson *et al.*, 2009; Hresko & Haga, 2012).

A performance-based model was tested by UnitedHealthcare in its approach to coverage of Genomic Health's Oncotype DX^®^. Oncotype DX breast cancer assay is a PGx test shown to aid in oncologists' decision-making on which patients with early breast cancer are most likely to benefit from chemotherapy (Dowsett *et al.*, 2010). Testing was expected to reduce inappropriate use of chemotherapy, but little data existed to support that assertion. UnitedHealthcare wanted evidence that they were getting sufficient value and cost offsets to warrant the coverage and reimbursement amount for the test. To help achieve this goal, an internal study was conducted on the proportion of women whose treatment choice coincided with the PGx test results (Carlson *et al.*, 2009). UnitedHealthcare monitored use of the test, and if clinicians did not take action based on the test results, the reimbursement amount was renegotiated to align with the actual value received.

Coverage and reimbursement models will likely continue to evolve to hold patients and health care providers more accountable for results of PGx testing. For these new models to work effectively, patient education is needed, as they will have to be involved in the testing and treatment paradigms and potentially incur costs based on these decisions. This is analogous to other areas where patients may elect to pay out-of-pocket when guidelines and insurance coverage are out-paced by advancing research/technology and perceived value in the marketplace. Mammogram reports are now required to include information on breast tissue density. Women with dense breast tissue are 4–5 times as likely to get breast cancer than women with low breast density (Yaghjyan *et al.*, 2011). However, there are no special recommendations or screening guidelines for women with dense breast tissue, and insurance generally does not cover more sensitive follow-up tests such as ultrasound or MRI. Women have the option of paying for more sensitive tests themselves.

### (iii) PGx is not the only factor influencing outcomes

While PGx testing may indicate a drug is safe or suitable for a patient, factors other than genetic polymorphisms have a significant effect on drug pharmacokinetics and pharmacodynamics. Epigenetic factors reveal a molecular basis for how information other than DNA sequence influences gene expression and can include epistatic interactions (Heil, 2013), patient age and gender, concomitant drug use and lifestyle factors (e.g., smoking, diet), which can all impact the results of therapy. Payers may be reluctant to provide coverage for a PGx test if these factors are not adequately addressed or controlled for. Therefore the integration of epigenetic, lifestyle and genetic influences are needed in order to interpret utility of PGx testing and to allow for adoption into current treatment practices and payer reimbursement. The specific data points of interest vary between payers and are still evolving, however, which adds to the challenges and research complexity. Efforts underway to address various aspects of these needs include the National Cancer Institute (NCI) program on the role of both pharmacoepidemiology and pharmacogenomics in treatment responses and adverse outcomes for oncology drugs (Freedman *et al.*, 2010) and Reaction Biology Corporation, which is creating a database of epigenetic drug interactions with a grant from the National Center for Advancing Translational Sciences (NIH, 2014).

### (iv) Ethical and social issues

The Genetic Information Nondiscrimination Act established in 2008 (NIH, 2015) is designed to protect individuals from discrimination based on genetic information. Ethical and social implications of genetic testing generally do not distinguish between PGx-guided therapy and testing for disease susceptibility. Consent for PGx testing designed to individualize drug therapy may not need the same level of scrutiny and requirements as genetic testing for disease susceptibility; however, as PGx testing continues to evolve, new research programs will emerge, such as the grant-backed NCI program addressing the ethical, legal, social and data-sharing implications of PGx and pharmacoepidemiologic research (NCI, 2014). A lessening in regulation and consent requirements for PGx testing might facilitate implementation as long as privacy and confidentiality are ensured for employment and payer coverage decisions (Dressler & Terry, 2009). This is an issue without current consensus.

### (v) Adoption of new technologies and standards

The factors and considerations previously discussed are important prerequisites for adoption of testing in the absence of a regulatory or legislative mandate. An additional, important consideration is the real and perceived complexity of PGx testing, including integration into existing workflows, interpretation of results and availability of information at the point of care. Research and prior examples have demonstrated that technologies such as PGx, which are complex and highly networked, are adopted more slowly. In sum, not only must the test be accurate, but it must be easy to use and access relative to the clinical problem at hand. Additional considerations include advances in technology that include Big Data initiatives such as the 100,000 Genomes Project and ‘Precision Medicine Intitiative’ that may play a role in genetic data management and interpretation, and ultimately impact stakeholder acceptance and utilization of PGx information.

## 6. Summary and conclusions

The adoption of PGx-guided therapy faces commercial, economic, educational and ethical barriers to integration into clinical practice and acceptance by practitioners, patients and payers. Acceptance of PGx testing varies considerably by therapeutic area. In some areas, such as oncology and cardiovascular disease, PGx testing is already utilized for selecting appropriate patients and/or establishing treatment and dosing guidelines, while in other areas such as antiplatelet therapy, the PGx approach has been mainly used for the identification, validation and development of new meaningful biomarkers, and is considered experimental by most stakeholders. The acceleration of adoption of precision medicine in general, and for PGx testing in particular, will be determined by how quickly robust evidence can be accumulated that shows a return on investment for payers in terms of real dollars, for clinicians in terms of patient clinical responses, and for patients in terms of economic, health and quality of life outcomes.
